# Essential Oil and Hydrophilic Antibiotic Co-Encapsulation in Multiple Lipid Nanoparticles: Proof of Concept and In Vitro Activity against *Pseudomonas aeruginosa*

**DOI:** 10.3390/antibiotics10111300

**Published:** 2021-10-26

**Authors:** Rayhane Ben-Khalifa, Frédéric Bustos Gaspar, Cristina Pereira, Leila Chekir-Ghedira, Soraya Rodríguez-Rojo

**Affiliations:** 1 Unit of Natural Bioactive Substances and Biotechnology UR17ES49, Faculty of Dental Medicine, University of Monastir, Monastir 5000, Tunisia; benkhalifarayhane@yahoo.com (R.B.-K.); leila.chekir@laposte.net (L.C.-G.); 2 Research Institute on Bioeconomy (BioEcoUVa), High Pressure Processes Group, School of Industrial Engineering, University of Valladolid, 47011 Valladolid, Spain; 3 iBET—Instituto de Biologia Experimental e Tecnológica, 2781-901 Oeiras, Portugal; cpereira@ibet.pt; 4 ITQB NOVA—Instituto de Tecnologia Química e Biológica António Xavier, Universidade Nova de Lisboa, 2780-157 Oeiras, Portugal

**Keywords:** multiple lipid nanoparticles, rosemary essential oil, cefepime, encapsulation, antimicrobial activity, *Pseudomonas aeruginosa*

## Abstract

In the worldwide context of an impending emergence of multidrug-resistant bacteria, this research combined the advantages of multiple lipid nanoparticles (MLNs) and the promising therapeutic use of essential oils (EOs) as a strategy to fight the antibiotic resistance of three *Pseudomonas aeruginosa* strains with different cefepime (FEP) resistance profiles. MLNs were prepared by ultrasonication using glyceryl trioleate (GTO) and glyceryl tristearate (GTS) as a liquid and a solid lipid, respectively. Rosemary EO (REO) was selected as the model EO. REO/FEP-loaded MLNs were characterized by their small size (~110 nm), important encapsulation efficiency, and high physical stability over time (60 days). An assessment of the antimicrobial activity was performed using antimicrobial susceptibility testing assays against selected *P. aeruginosa* strains. The assays showed a considerable increase in the antibacterial property of REO-loaded MLNs compared with the effect of crude EO, especially against *P. aeruginosa* ATCC 9027, in which the minimum inhibitory concentration (MIC) value decreased from 80 to 0.6 mg/mL upon encapsulation. Furthermore, the incorporation of FEP in MLNs stabilized the drug without affecting its antipseudomonal activity. Thus, the ability to co-encapsulate an essential oil and a hydrophilic antibiotic into MLN has been successfully proved, opening new possibilities for the treatment of serious antimicrobial infections.

## 1. Introduction

Antibiotic resistance has become a major health problem worldwide, leading to serious consequences in the treatment of infectious diseases [1]. High intrinsic resistance to several antimicrobial agents is exhibited by many microorganisms especially *Pseudomonas aeruginosa*, an important opportunistic microorganism commonly found in hospitals. Increasing acquired resistance mechanisms in this pathogen has led to treatment failure of nosocomial respiratory tract infections [2,3,4] and a significant increase in attributable mortality among patients [5].

The emergence of multidrug-resistant phenotypes in *P. aeruginosa* was shown to be correlated with a reduced permeability of the outer membrane, efflux mechanisms such as the downregulation of porin synthesis [6], and the synthesis of enzymes such as AmpC β-lactamases able to promote the degradation of many antimicrobials. Particularly, resistance to carbapenems caused by low membrane permeability and/or efflux pumps is frequently observed in these multi-drug resistant strains. Cefepime (FEP), a fourth-generation zwitterionic cephalosporin, is one of the few remaining antibiotics that has a reliable action against *P. aeruginosa*, although increasing resistance to FEP among different species of *Pseudomonas* has been previously reported [7].

The hope of overcoming the threat of bacterial resistance via the development of new antibiotics is reduced since pathogens tend to quickly develop resistance to novel antimicrobial molecules [8]. It is therefore imperative that existing antibiotics be used more thoughtfully. In this regard, natural compounds with intrinsic antimicrobial potential such as polyphenols, alkaloids, or sulfur-containing compounds [9,10,11] as well as essential oils (EOs) [12,13,14] have been proposed as a viable complement to fight the problem of antibiotic resistance. Several studies reported that, due to the complexity of their chemical constituents, EOs are able not only to exhibit powerful antimicrobial activity but also to act in synergy when simultaneously used with conventional antibiotics, therefore enhancing their antimicrobial efficacy [15,16,17]. The major compound of *Rosmarinus officinalis* (rosemary) EO (REO), 1,8-cineole, can modify the permeability of the bacterial cytoplasmic membrane, can enhance antibiotic intake, and can destroy the physical structure of the membrane in MRSA strains and Gram-negative bacteria [18,19]. In fact, REO has been used in traditional medicine all around the world as an antiseptic and for the treatment of respiratory diseases, among other illnesses [20].

Despite the described biological and medicinal properties of EOs, their industrial applications are very limited due to their lipophilicity, high volatility, and easy decomposition after exposure to environmental factors [21]. In this context, the nano-encapsulation of EOs has been suggested as a promising strategy to enhance their water solubility, to improve their bioavailability, and to preserve their physicochemical stability during processing and storage [22].

The use of lipid carriers has been considered an alternative approach to enhancing the antibacterial propriety of numerous antimicrobial agents. In fact, it was demonstrated that the lipid layers of these types of systems are able to fuse with the bacterial outer membrane, allowing for the direct administration of encapsulated drugs [23,24,25]. Nanostructured lipid carriers (NLCs) are an example of second-generation lipid nanoparticles composed of a mixture of solid and liquid lipids surrounded by a surfactant. The liquid lipid creates imperfect lipid crystals, allowing for a higher loading of bioactive molecules and increasing the stability upon storage [26]. Recent investigations revealed the effectiveness of NLCs in successfully encapsulating REO [27]. However, owing to the hydrophobic nature of the lipid matrix, NLC formulations are more appropriate for incorporating lipophilic rather than hydrophilic agents [28]. Thus, a new generation of lipid nanoparticles, called multiple lipid nanoparticles (MLNs), has been developed, combining the advantages of double emulsions and NLCs [29].

To the best of our knowledge, no previous reports examining the use of EOs and antibiotics co-loaded MLNs have been described. Hence, this study has a double aim. First, to demonstrate the capability to load a hydrophobic (REO) and/or hydrophilic component (FEP) in MLNs. Second, to test in vitro the loaded (REO-MLNs and FEP-MLNs) and co-loaded (REO-FEP-MLNs) formulations prepared under selected conditions against *P. aeruginosa*. 

## 2. Results and Discussion

### 2.1. REO Characterization

The major components identified and quantified in REO by GC–MS were 1,8-cineole (23.8%), α-pinene (16.2%), camphor (15.3%), and camphene (9.8%), as shown in Table 1. According to the present chemical composition, it can be confirmed that REO corresponds to the Spanish chemotype (18% to 26% of 1,8 cineole) [30,31].

### 2.2. MLN Preparation and Characterization

The quality and the stability of colloidal systems are affected by various parameters, particularly the preparation procedures and the nanoparticles composition, in this case, MLNs. The particle size distribution (PSD), the stability measured by zeta-potential (ZP), and the percentage of encapsulation efficiency of the active component (EE (%)) are useful measurements for the evaluation of MLN physicochemical properties.

#### 2.2.1. Effect of MLN Composition on REO Encapsulation

The most critical variables related to MLN composition are (1) the oil phase composition, which is the percentages of EO, liquid lipid (glyceryl trioleate, GTO), and solid lipid (glyceryl tristearate, GTS); (2) the water-to-oil-phase mass ratio in the first emulsion (W1:O); and (3) the mass ratio of this first emulsion to the outer inner phase ((W1:O): W2). Their effects in the response element PSD, determined as the mean particle size (d(3,2)) and span; ZP; EE (%); and loading capacity (LC) of REO were analyzed in Table 2 for 11 emulsions prepared with a constant concentration of the oil phase surfactant (10% Pluronic^®^ L64) as well as the aqueous surfactant in W2 (5% Tween^®^ 80).

In the first set of experiments (MLN 1 to 6), the percentage of GTO in MLNs varied from 0 to 80%, while the amount of REO varied oppositely and all other parameters were kept constant. As presented in Table 3, the results revealed a nanometric and uniform size distribution of the obtained MLNs. A typical PSD curve was shown in Figure S1 (Supplementary Materials). By increasing the content in GTO, the PSD slightly increased from 101 to 112 nm in terms of average size diameter and from 0.9 to 1.2 in terms of span, although this variation is not statistically significant. Similarly, previous work performed on NLC systems did not show significant differences between the tested formulations prepared with different cardamom-EO-to-olive-oil ratios [32]. The ZP measurements revealed a negative surface charge of the examined MLNs. The values ranged from −40 to −58 mV, confirming the good physical stability of the developed MLNs (Figure S2, Supplementary Materials). By increasing the GTO percentage, no remarkable increasing trend was noted. On the contrary, it was shown that the EE (%) of REO diminished significantly when the amount of GTO in the formulations increased. The highest value was obtained in MLN 1, containing only REO as the liquid lipid component of the oil phase. Similar results were found in the study of Keivani et al. (2018) [32], who demonstrated that the increase in cardamom EO content led to a high EE (%). To explain such an observation, they associated that tendency with the lipophilic nature of the EO used. In fact, the dissolution profile of the core material in the lipid matrix is considered a determining parameter when the EE is evaluated: the more the incorporated ingredient is liposoluble, the more it is partitioned into the lipid matrix, which results in a higher EE [33]. In this work, the high required hydrophilic–lipophilic balance of REO (required HLB = 15) [34] may be responsible for its limited solubility in GTO and, thus, for the moderate EE (%) of REO in GTO-rich MLNs. 

In the second series of experiments (MLN 5 and 7 to 9), the ratio of solid to liquid lipids varied. The percentage of GTS and GTO ranged from 0 to 30% and from 40 to 70%, respectively, while the REO content was kept constant (20%). As shown in Table 2, the increase in GTS content from 0 to 20% lead to a slight increase in PSD. However, when it reached 30%, a clear increase in mean particle size was observed (from 110 to 219 nm). Likewise, the width of droplet size distribution was noticeably enlarged: the span varied from 0.9 to 2.0. Similar findings were recorded in the literature [35,36]. A possible explanation for this fact could be the excess solid lipid in MLNs which increases the melting point of the mixture promoting lipid crystallization at ambient temperature [37]. Additionally, a high solid lipid content in lipid carrier contributes to the lofty viscosity in the dispersed phase and therefore contributes to more resistant emulsions against shear forces of emulsification; thus, bigger particles are formed [38]. Concerning EE calculations, no considerable variation was noted upon increasing the GTS proportion from 0 to 30%, presenting the effect of the initial content of REO. With this set of experiments, no linear correlation was observed between the ZP and the percentage of GTS used. A similar trend was noted in the work of Shah and co-workers [39]. As presented in Table 2, MLN 7 (0% GTS-70% GTO) showed the highest absolute value (−66 mV). The probable reason for this finding is the absence of solid lipid, which contributed to the formation of a stable emulsion of small droplets. Moreover, it is reported that a high value of ZP could be related to the ionized fatty acids in GTO, which provide a high negative charge and produce an electric barrier on the surface of particles [40,41]. Accordingly, the system resisted aggregation, showing good physical stability [42].

Finally, the mass ratio W1:O in the primary emulsion as well as the ratio of (W1:O) to W2 varied while the composition of the oil phase remained unaltered (MLN 5, 10, and 11). As shown in Table 2, no notable differences in the PSD and ZP values of MNL 5 and 11 were found, which is similar to what was noted by Min and collaborators [43]. In fact, the use of lipophilic and hydrophilic emulsifiers in constant proportions (10% and 5%, respectively) to prepare diverse formulations with distinct W:O:W ratios could conserve the physicochemical properties of the prepared nanoparticles; hence, similar MLNs were formed.

#### 2.2.2. Effect of MLN Composition on FEP Encapsulation

To investigate the influence of the liquid component percentages in the oil phase (GTO and REO) in FEP formulation performance (PSD, FEP-EE (%), and FEP-LC), MLN 1, 3, and 6 were selected using a constant concentration of 10 mg/mL of FEP solution in phosphate buffer (W1). The composition of GTS was kept constant since, according to previous results (Table 2), an increment in GTS content could produce a significant rise in particle size. Additionally, the effect of increasing the global content of the oil phase with respect to W1 and W2 in the FEP formulation performance was studied only through MNL 11, since MLN 10 formulation previously proved to be unstable (Table 2).

As shown in Table 3, the addition of FEP does not affect the PSD of the particles compared with those containing only EO (Table 2), with d(3,2) values between 102 and 125 nm, in agreement with illustrative images by transmission electron microscopy (Figure S3, Supplementary Materials). The FEP-EE (%) values, ranging from 10 to 32%, indicated the potential capacity of the MLN formulations to entrap hydro-soluble drugs. Using classic lipid carriers such as liposomes for FEP encapsulation, low EE (%) values of 2% and 3% were previously obtained [44,45]. They associated the observed results with the partition coefficient of FEP, which favors high content leakage of the drug to the aqueous phase [45]. In this case, the use of MLN formulations, characterized by a complex and rigid lipid matrix, is recommended for the better loading of water-soluble compounds. Moreover, no remarkable variation in FEP-EE was demonstrated when GTO quantity was increased within nano-dispersions. This finding can be explained based on the low affinity of hydrophilic drugs, such as FEP to the oil phase. GTO with a low HLB value (HLB = 0.8) has excessive hydrophobicity, which allows FEP to mainly reside in the inner aqueous phase (W1) and not in the lipid phase; thus, the drug-to-lipid ratio does not affect the encapsulation behavior of FEP to a large extent. In this study, MLN 6 showed the lowest EE (%), probably due to the high value of the span, which favors particle coalescence and leads to FEP expulsion to the external aqueous phase (W2). Generally, the EE (%) values achieved here are similar or slightly higher than the encapsulation of a hydrophilic antiretroviral agent, lamivudine (3TC), in the MLNs (EE = 20% ± 2%) in a previous work by Cavalcanti and collaborators [29].

The mass ratio between the different phases is a determinant parameter that could directly influence the drug encapsulation in MLN dispersions. When evaluating the effect of W1:O and (W1:O):W2 ratios variation, no significant change in PSD was detected. However, by increasing this ratio, FEP-EE (%) considerably decreased. Indeed, as seen in Table 3, increasing the amount of water with respect to the lipid phase (1.6:2.4 in the first emulsion and 4:6 in the second one), MLN 11 was able to encapsulate only 10% of FEP. A similar trend was observed in the results of Yildirim et al. [46] and Ramesh [47], who noticed that the EE was reduced when the internal or the external aqueous phases increased. This could be due to the partitioning or diffusion of the inner FEP into the external aqueous phase (W2). In fact, it is interesting to note that the ratio of oil to W1 was 4 in MLN 1, 3, and 6, and only 1.5 in MLN 11. This means that there was more oil to cover the inner phase for similar particle sizes in the first case; hence, the barrier to avoid FEP leakage to W2 was thicker, leading to better drug entrapment in MNL 1, 3, and 6.

The MNL 3 formulation was selected to study the effect of the initial FEP concentration in the inner aqueous phase (W1) in the 2 to 30 mg/mL range. As expected, the results indicated that, as the concentration of FEP in W1 increases, the FEP-LC increases (Table S1, Supplementary Materials) with similar values of FEP-EE (%) due to the high solubility of FEP in the aqueous media and its low lipophilicity.

### 2.3. Antimicrobial Susceptibility Testing (AST) Assays 

#### 2.3.1. AST Assays of Pure Compound

The antibacterial potential of REO and FEP for the three strains selected was determined using the AST assay to obtain minimum inhibitory concentration (MIC) values of the compound required to prevent bacterial growth. The results are summarized in Table 4. 

Based on the MIC values presented in Table 4 and according to the interpretation of the breakpoint values presented in the performance standards for antimicrobial susceptibility testing [48] regarding FEP, *P. aeruginosa* PT3087 is classified as intermediate (8 < MIC < 32 µg FEP/mL) and both *P. aeruginosa* ATCC 9027 and *P. aeruginosa* PS16 are classified as susceptible (MIC ≤ 8 µg FEP/mL).

When REO was assayed, the obtained MIC results showed that it exhibited an inhibitory effect against *P. aeruginosa* PT3087 at 40 mg/mL. However, the minimum concentration needed to inhibit *P. aeruginosa* ATCC 9027 and *P. aeruginosa* PS16 was 80 mg/mL. The REO potential to inhibit the growth of *P. aeruginosa* has been well assessed [49,50]. The results displayed in this work are similar to those demonstrated by Saviuc et al. [51], who reported a MIC range from 13 to 52 mg/mL against the tested *P. aeruginosa*. The mechanism of action of REO to inhibit bacterial growth is still unclear, although using flow cytometry, some studies demonstrated that REO could act by inducing cellular wall permeabilization and active inhibition of efflux pumps [51].

#### 2.3.2. AST Assays of Loaded MLNs

The active compound concentration of selected formulations for AST assay are shown in Table 5. The global composition and oil phase composition are those of MLN 3 for REO and REO-FEP formulation. This MLN composition was selected since it is the one allowing for a higher REO loading capacity while having liquid and solid lipid (GTO and GTS) in the oil phase (Table 2). Additionally, it allows for a higher FEP-EE (%) (Table 3). The MLN for FEP formulation is that of MLN 6 since it is comparable with MLN 3, but in this case, REO is replaced by an additional amount of GTO. For the FEP formulation, an initial concentration of 2 mg/mL in W1 was used, corresponding to a total dose of 80 μg FEP/mL of formulations, due to the low MIC values of free FEP against examined strains (Table 4) in comparison with the LC and also considering that non-encapsulated FEP was present in the formulation. For REO, the LC values (Table 2) are similar to the MIC values of free REO (Table 4); further, the non-encapsulated amount of REO was assumed to be lost during production.

Additionally, a REO-free and FEP-free formulation was also tested to evaluate the antimicrobial effects of the lipid components, GTO, and GTS. The composition of the oil phase of this empty formulation was that of MLN 6. Since the antibacterial effect of these lipids was expected to be limited, only one empty formulation was tested.

The MIC values of each MLN formulation as well as its concentration in the active compound (REO and/or FEP) at the corresponding dilution in cation-adjusted Mueller–Hinton broth (CAMHB) growth media are displayed in Table 6.

Tests with the unloaded formulation, MLN 6 (empty), showed that the lipids forming the MLN exhibited slight antimicrobial activities against *P. aeruginosa* ATCC 9027 and *P. aeruginosa* PS16 at 62.5 and 125.0 µL/mL, respectively (Table 6). However, no activity was detected against *P. aeruginosa* PT3087 (MIC > 500 µL/mL). The observed antibacterial activity against sensitive strains could be related to the ability of some lipids to destabilize the phospholipid membrane of bacterial cells [52].

In contrast, the antipseudomonal effect of REO-loaded MLNs was greater than pure EO against all tested bacteria; the amount of REO needed to achieve the MIC, independently of the strain tested, is always lower when REO is encapsulated (Table 6) compared with REO alone (Table 4). The MIC value reduction varies from 18-fold to more than 100-fold. The observed enhancement of inhibitory effects against all tested strains suggests that loading EOs into nanoscale particles improves their bacteriostatic activity considerably. Our results agree with previous studies that showed that the antimicrobial properties of encapsulated EOs were either better or equivalent to their native form. For example, compared with free oil, the nano-encapsulation of *Thymus daenensis* EO improved its antibacterial effect against *E. coli* [53]. Furthermore, the formulation of anise myrtle and lemon myrtle EOs in nanoemulsions resulted in enhanced antibacterial potential compared with lemon myrtle EO alone; however, no improvement on anise myrtle EO activity was shown [43]. Additionally, using solid lipid nanoparticles to encapsulate *Eugenia caryophyllata* EO, Fazly et al. [54] demonstrated that these formulations presented higher antibacterial activities compared with the native oil. The mechanism by which the nano-encapsulation of EOs enhances its antibacterial activity is not fully understood. One possible explanation is associated with nanoparticle size. In fact, it was demonstrated that small droplets of nanoemulsions can bring encapsulated EOs closer to the surface of the bacterial membrane and improve their accessibility [53]. Furthermore, the similarity of structure between lipid nanocarriers and the bacterial cell membrane favors the passive cellular absorption of antibacterial agents probably by disrupting the integrity of the phospholipid bilayer, releasing the active compounds into the medium [55,56]. Nasseri and co-workers [57] suggested that the enhancement of the antimicrobial activity of *Zataria multiflora* EO-loaded SLNs may be due to their spherical shape, which provides the longest route for the movement of EOs within the nanoparticles and the lowest contact area with the aqueous phase.

For FEP formulations, the MIC values are similar to those of free FEP, as displayed in Table 4 and Table 6, respectively. Our results are in accordance with the research of Moyá et al. [44], who found no marked difference between MICs of free and liposomal FEP against *E. coli* strains. In contrast, when liposomes or SLNs were used to encapsulate different antibiotics (e.g., ciprofloxacin, meropenem, and amikacin), other reports found that the encapsulated forms presented significantly lower effectiveness against *P. aeruginosa* than free antibiotics [24,58,59]. Interestingly, loss of activity of aqueous FEP solutions, stored at different temperatures (4 °C, 21 °C, 37 °C, and 42 °C) was observed (Table S2, Supplementary Materials), while FEP-loaded MLNs, kept at 4 °C, were able to stabilize the antibiotic without affecting its antibacterial property for at least two months. Thus, MLNs could be used to stabilize labile compounds (such as FEP) in various biological and pharmaceutical applications.

According to co-loaded MLNs, a clear decreasing trend in the REO amount needed to achieve the MIC was recorded in MLN 3 (FEP + REO) compared with the dose of pure oil, which showed bacterial inhibition against both susceptible and resistant strains (Table 6). The maximum MIC reduction (67-fold) was observed in *P. aeruginosa* ATCC 9027. Nonetheless, it is important to note that such a reduction was greater in MLN 3 containing only REO. In contrast, the MIC values of co-encapsulated FEP were higher than that of free FEP. The observed decrease in the antibacterial activity could be explained by an antagonistic action resulting from the REO-FEP combination. Recently, studies conducted to assess if combinations including EOs and FEP are able to increase the antibiotic susceptibility of *P. aeruginosa*, among other bacteria, have been reviewed [60]. However, no previous research assessed the combined action of REO and antibiotics. An antagonistic effect has been observed for the combinations of sage (*Salvia officinalis*) EO and FEP against *P. aeruginosa* ATCC 9027, where 1,8-cineole (29%) was the major oxygenated terpene [61]. Terpenoid ethers, such as 1,8-cineole, have been formerly reported to achieve a lower capacity to sensitize pathogens to antibiotic activity as they lack free hydroxyl groups, a structural feature present in both phenolic and alcoholic terpenoids [61,62]. Nevertheless, REO showed a synergistic effect in combination with aminoglycoside antibiotics against *E. coli* [60].

## 3. Materials and Methods

### 3.1. Materials

The lipids, GTO and GTS; the non-ionic surfactants (Pluronic^®^ L64 and Tween^®^ 80); and FEP were all purchased from Sigma-Aldrich (Madrid, Spain). REO was purchased from COCOPE S. Cooperativa, Peñafiel, Spain. Its main composition was identified by gas chromatography–mass spectrometry (GC–MS), as described in detail in Section 3.3.4.

Ultrapure water, used to prepare the formulations, was produced by a Milli-Q unit from Millipore (Burlington, MA, USA). The chloroform used for encapsulation efficiency determination was from TCI Europe N.V. (Zwijndrecht, Belgium), and the acetonitrile used for HPLC analysis was from Panreac Quimica SLU (Barcelona, Spain). The phosphate-buffered solution was prepared using disodium hydrogen phosphate heptahydrate (Na_2_HPO_4_·7H_2_O) and sodium dihydrogen phosphate monohydrate (NaH_2_PO_4_·H_2_O) from Panreac Quimica SLU (Barcelona, Spain). All chemicals were used as provided.

Three strains of *P. aeruginosa* were used in this study as targets for the AST assays. The susceptible strain, *P. aeruginosa* ATCC 9027, was obtained from ATCC (American Type Culture Collection). Two multidrug-resistant strains were also used: *P. aeruginosa* PS16 (resistant to amikacin, ciprofloxacin, imipenem, meropenem, and tobramycin) is a nosocomial strain isolated at University Hospital of Fattouma Bourguiba in Monastir, Tunisia, and *P. aeruginosa* PT3087 (resistant to ciprofloxacin, gentamicin, imipenem, levofloxacin, meropenem, piperacillin-tazobactam, and tobramycin) is also a nosocomial strain but was isolated from a patient in Portugal. *P. aeruginosa* ATCC 27853 was used as a quality control strain to validate the FEP stock solution and the general performance of the assay.

CAMHB (BD Difco, Sparks, NV, USA) was used as a growth medium for AST assays.

### 3.2. MLN Preparation

MLNs were prepared using the ultrasonication technique as described elsewhere [29], with some modifications. In brief, the solid lipid (GTS) was melted in the liquid lipid (GTO), previously mixed with 10% Pluronic^®^ L64, by heating at 85 °C for 15 min. REO was added to the lipid phase at the end of the melting process to prevent the loss of volatile compounds. Subsequently, the first aqueous phase (W1), heated at 85 °C, was added dropwise to the oil phase. The mixture was homogenized by magnetic stirring at 1500 rpm for 3 min followed by probe-type sonication (Vibra cell 75185, 130 w, 20 Hz) for 20 min with an amplitude of 90%. Subsequently, the aqueous solution of 5% Tween^®^ 80 (W2, heated at 85 °C) was added to the first prepared emulsion (W1/O). The mixed solution was again homogenized by ultrasounds for 15 min at the same amplitude to produce the double emulsion (W1/O/W2). The final formulation was cooled down at room temperature to obtain the MLN dispersion.

Screening tests were performed to determine some parameters such as sonication time (Figure S5, Supplementary Materials) and amplitude (Table S3, Supplementary Materials) for each emulsification step, and the emulsifier concentration in the lipid phase and in the outer aqueous phase (Table S4, Supplementary Materials).

The lipid composition as well as the ratio between phases of different tested MLNs can be found in Table 2.

FEP encapsulation was carried out by dissolving a predetermined amount of this drug in phosphate-buffered solution (pH 6, 0.1 mol/L) [63]. The obtained solution was used as the inner aqueous phase (W1) of the primary emulsion. All of the performed assays evaluating the effect of lipid composition on FEP encapsulation of the tested formulations (MLN 1, 3, 6, and 11) were prepared at a FEP concentration of 10 mg/mL in W1. Likewise, the influence of FEP concentration in W1 (2, 10, 20, and 30 mg/mL) on FEP-EE in MLN 3 was investigated.

### 3.3. MLNs Characterization

#### 3.3.1. PSD

The PSD of freshly prepared MLNs was determined using laser diffraction (Mastersizer 2000, Malvern Instruments, Worcestershire, UK). The size measurement range of this equipment is 0.02–2000 μm, using a diode laser of 4 mW with a dual wavelength detection system with red (633 nm) and blue (436 nm) lights. The sample was diluted with deionized water in the dispersion unit (Hydro SM) to obtain an adequate level of laser obscuration to prevent multiple scattering effects. The particle size measurement is reported as the average diameter in the volume distribution (d(3, 2)), and the distribution width was expressed in terms of span, which is calculated as follows:

(1) $$\mathrm{SPAN}=\frac{d_{90}-d_{10}}{d_{50}}$$

where d_10_, d_50_, and d_90_ are defined as the maximum particle size for a given percentage volume of the sample: 10%, 50%, and 90%, respectively. By monitoring these three parameters, it is possible to determine if there are significant changes in the main particle size as well as changes at the extremes of the distribution, which could be due to the presence of fine or oversized particles and agglomerates.

#### 3.3.2. MNL Stability: ZP Measurement

The ZP is related to the respective surface charge of particles; it is a useful parameter to predict the physical stability of colloidal systems. This parameter was determined using a Zetasizer Nano Z (Malvern Instruments, Worcestershire, UK) at 25 °C. Before the measurements, all samples were appropriately diluted (1:1000) with ultra-purified water. Each sample was analyzed at least in triplicate.

Additionally, the physical stability of MLN 5 was evaluated over 60 days at 4 °C following the evolution of PSD parameters, as shown in Section 3.3.1.

#### 3.3.3. REO-EE and REO-LC

The real concentration of REO in MLNs was determined using a direct method, as described below. In brief, a known amount of nanoparticles suspensions was first diluted (1:3) in water and then separated by gradient ultracentrifugation (271,000× *g*) at 4 °C for 1 h using Optima TM L-100XP Ultracentrifuge (Beckman Coulter Inc, Brea, CA, USA). The lipid supernatant was carefully removed, and 0.07 g of it was dissolved in 1.5 mL of chloroform (with 1% trans-2-hexenal as an internal standard) in an ultrasonic bath for 15 min. The obtained solution was further vortexed and filtered (PTFE, 0.22 µm), and the REO amount was analyzed by gas chromatography coupled with mass spectrometry (GC–MS), as detailed in Section 3.3.4.

The EE (%) and the LC of REO were calculated using the following equations:

(2) $$\mathrm{REO}\text{-}\mathrm{EE}\;\left(\%\right)=\frac{\mathrm{Real}\;\mathrm{concentration}\;\mathrm{of}\;\mathrm{encapsulated}\;\mathrm{REO}}{\mathrm{Theoretical}\;\mathrm{concentration}}\times 100$$

(3) $$\mathrm{REO}\text{-}\mathrm{LC}\;\left(\frac{\mathrm{mg}}{\mathrm{mL}}\right)=\frac{\mathrm{mass}\;\mathrm{of}\;\mathrm{encapsulated}\;\mathrm{REO}}{\mathrm{Total}\;\mathrm{volume}\;\mathrm{of}\;\mathrm{MLN}}$$

#### 3.3.4. GC-MS

The EO analysis was performed using a gas chromatographer (Agilent 6890) with a mass selective detector (Agilent 5973) from Agilent Technologies (Palo Alto, CA, USA) and an Agilent HP-5ms Capillary GC column. Helium was the carrier gas at 0.7 mL/min. The samples (1 µL) were injected at 250 °C in split mode (200:1). Pure REO was analyzed with a 500:1 split ratio. The oven temperature was programmed as follows: 5 min at 40 °C and 3 °C/min to 300 °C. Identification of the compounds was based on mass spectra identification with “Wiley Mass Spectral Library”.

#### 3.3.5. FEP-EE and FEP-LC

The FEP-EE (%) was determined using an indirect method [45]. The aqueous supernatant obtained by ultracentrifugation was filtered (nylon, 0.2 µm) and analyzed by HPLC. The HPLC was equipped with an isocratic pump (Waters 1515), an auto-injector (Waters 717), and an UV-Vis detector (Waters 2487) at the wavelength of 254 nm. The separation was performed with a C18 column 5 μm, 250 mm × 4.6 mm (Mediterranean Sea C18, Teknokroma, Barcelona, Spain). The mobile phase consisted of acetonitrile and water (10:90, *v/v*) with a flow of 1 mL/min. The column was maintained at 30 °C in the column oven. The injection volume was 20 µL.

The results were evaluated based on a linear calibration prepared with reference standard solutions of FEP dissolved in phosphate buffer (pH = 6) at different concentrations ranging from 10 to 100 ppm. 

The EE (%) as well as the LC of FEP were determined based on the following equations:

(4) $$\mathrm{FEP}\text{-}\mathrm{EE}\;\left(\%\right)=\frac{\mathrm{Total}\;\mathrm{amount}\;\mathrm{of}\;\mathrm{FEP}-\mathrm{Free}\;\mathrm{amount}\;\mathrm{of}\;\mathrm{FEP}}{\mathrm{Total}\;\mathrm{amount}\;\mathrm{of}\;\mathrm{FEP}}\times 100$$

(5) $$\mathrm{FEP}\text{-}\;\mathrm{LC}\left(\frac{\mu g}{\mathrm{mL}}\right)=\frac{\mathrm{mass}\;\mathrm{of}\;\mathrm{encapsulated}\;\mathrm{FEP}}{\mathrm{Total}\;\mathrm{volume}\;\mathrm{of}\;\mathrm{MLN}}$$

### 3.4. AST Assay

AST assays were performed according to the broth micro-dilution method described in the CLSI M07-A10 guidelines [63]. REO and FEP stock solutions as well as the formulations were dissolved in CAMHB without supplementation with emulsifiers.

In brief, the selected target samples (REO, FEP, and MLNs) were dispensed in a 96-well round-bottom microtiter plate and two-fold serially diluted in CAMHB. The *P. aeruginosa* inocula were prepared to reach a target bacteria concentration of 5 × 10^4^ CFU per well after standardization. A positive control (CAMHB and diluted inoculum), a medium sterility control (uninoculated CAMHB), and a sample sterility control (uninoculated 2-fold stock solution in CAMHB) were also performed. The plates were incubated under aerobic conditions at 37 °C for 18 h (±2 h). The MIC values were read as the lowest concentration of the antimicrobial solution at which visible growth was inhibited after incubation. In the case of sample opacity or an ambiguous MIC value reading, the cell viability reagent PrestoBlue (Invitrogen, San Diego, CA, USA) was used. The results were expressed as a median of MIC values obtained for each of the three biological replicates performed.

The FEP master stock solution was prepared at a concentration of 512 µg/mL in phosphate buffer, pH 6.0, and stored frozen until needed. The FEP working solution was diluted so that the first concentration of the range tested was 256 µg/mL. The REO stock solutions were freshly prepared by weighing a defined volume of REO and then adding the necessary amount of CAMHB so that the first concentration of the range tested was 160 mg/mL. Similarly, the formulation stock solutions were freshly prepared by adding a known volume of formulation into a defined volume of CAMHB medium so that the highest concentration tested was 500 µL/mL.

### 3.5. Statistical Analysis

The data obtained from MLN characterization was processed using the software SPSS (version 22) by one-way analysis of variance followed by the Tukey test. *p* < 0.05 was accepted as statistically significant in all cases. The data were expressed as mean ± standard deviation values of at least three independent experiments.

## 4. Conclusions

This study reports the successful preparation of REO- and FEP-loaded MLNs as well as its co-encapsulation for the first time. MLNs were prepared by a two-steps ultra-sonication process using a solid lipid (GTS), a liquid lipid (GTO), a lipophilic surfactant (Pluronic^®^ L64), and a hydrophilic emulsifier (Tween^®^ 80). The developed REO/FEP-loaded MLNs and co-loaded MLNs were characterized by their small size (≈100 nm), high homogeneity and stability (ZP ≥ −40 mV), as well as good EE (%).

Based on the AST results, the formulation of REO in nanoscale particles greatly enhanced its antibacterial activity against all tested strains of *P. aeruginosa*. A higher MIC reduction, up to 100-fold, was shown against strains of *P. aeruginosa* sensitive to FEP (ATCC 9027 and PS 16), while an interesting 9-fold MIC reduction was noted in the case of the intermediate *P. aeruginosa* PT3087 strain when treated with REO-MLNs. On the other hand, the FEP-loaded formulations were as effective as the free drug.

Thus, the possibility to co-load in MLNs a lipophilic ingredient, EO, and a hydrophilic ATB has been demonstrated. This dual delivery system should be further studied for use in pharmaceutical and medical applications as an effective approach to fight the disturbing rise in antibiotic resistance in bacteria. Nevertheless, before using this nanoscale formulation, all requirements should be met according to the existing regulations and directives of medical governing bodies and agencies.

## Figures and Tables

**Table 1:** Main components of REO analyzed by GC–MS.

Component	% Area	Retention Time (min)
α-pinene	16.2	9.07
camphene	9.8	9.55
β-pinene	6.0	10.58
1,8-cineole	23.8	12.99
camphor	15.3	21.06
borneol	3.5	22.72
2-pinen-4-one	1.6	25.40
bornyl acetate	4.9	29.55
trans-caryophyllene	2.4	35.62

**Table 2:** Effect of mass phase ratios and lipid composition on MLN physicochemical properties.

	Global Composition		Oil Phase Composition			Results				
MLN#	W1:O (*w:w*)	(W1:O):W2 (*w:w*)	GTO (%)	GTS (%)	REO (%)	PSD		ZP (mV)	REO-EE (%)	REO-LC (mg/mL)
						d(3,2) (nm)	Span (-)			
**1**	0.4:1.6	2:8	0	10	80	103 ± 4 ^a^	0.9 ± 0.1 ^a^	−40 ±3 ^d^	51.2 ± 0.8 ^a^	65.3 ± 1.3 ^a^
**2**			10		70	99.5 ± 0.7 ^a^	0.829 ± 0.007 ^a^	−55 ± 6 ^b^	44.2 ± 1.1 ^b^	49.3 ± 1.1 ^b^
**3**			20		60	101 ± 3 ^a^	0.8 ± 0.1 ^a^	−42 ± 5 ^d^	44 ± 3 ^b^	42 ± 3 ^c^
**4**			40		40	109 ± 4 ^a^	0.843 ± 0.002 ^a^	−58.1 ± 0.7 ^b^	43 ± 4 ^bc^	27 ± 2 ^d^
**5**			60		20	111 ± 3 ^a^	0.9 ± 0.1 ^a^	−48 ± 3 ^c^	31 ± 7 ^d^	10 ± 2 ^f^
**6**			80		0	112 ± 3 ^a^	1.18 ± 0.04 ^a^	−55 ± 4 ^b^	-	-
**7**			70	0		110 ± 4 ^a^	0.90 ± 0.10 ^a^	−66 ± 4 ^a^	31.3 ± 0.6 ^d^	9.9 ± 0.3 ^f^
**8**	0.4:1.6	2:8	50	20	20	115.5 ± 0.7 ^a^	0.92 ± 0.03 ^a^	−44 ± 2 ^cd^	39 ± 2 ^bc^	12.5 ± 0.6 ^f^
**9**			40	30		219 ± 82 ^b^	2.0 ± 0.4 ^b^	−58 ± 2 ^b^	31 ± 6 ^d^	10 ± 2 ^f^
**10**	0.9:2.1	3:7	60	10	20	183 ± 92 ^ab^	27 ± 4 ^c^	-	-	-
**11**	1.6:2.4	4:6	60	10	20	107.5 ± 0.7 ^a^	1.194 ± 0.005 ^a^	−43.2 ± 0.9 ^cd^	37 ± 2 ^cd^	18.3 ± 1.1 ^e^

Different lowercase letters (a–f) indicate statistically significant differences, *p* < 0.05, in each column.

**Table 3:** Effect of mass phase ratios and lipid composition on REO-FEP- and FEP-MLN physicochemical properties.

	Global Composition		Oil Phase Composition			Results			
MLN#	W1:O *(w:w*)	(W1:O):W2 (*w:w)*	GTO (%)	GTS (%)	REO (%)	PSD		FEP-EE (%)	FEP-LC (μg/mL)
						d(3,2) (nm)	Span (-)		
**1**	0.4:1.6	2:8	0	10	80	110 ± 7 ^ab^	0.8 ± 0.2 ^a^	32 ± 2 ^c^	128 ± 8 ^a^
**3**			20		60	102 ± 5 ^a^	0.82 ± 0.03 ^a^	27 ± 8 ^bc^	108 ± 32 ^a^
**6**			80		0	111 ± 2 ^b^	1.62 ± 0.03 ^b^	22 ± 3 ^b^	88 ± 11 ^a^
**11**	1.6:2.4	4:6	60	10	20	125 ± 21 ^ab^	1.3 ± 0.4 ^ab^	10 ± 1 ^a^	160 ± 16 ^b^

Different lowercase letters (a–c) indicate statistically significant differences, *p* < 0.05, in each column.

**Table 4:** MIC values of REO and FEP. MIC values are represented as the median of three independent assays.

*P. aeruginosa* Target Strains	MIC_median_ (MIC_n=1_/MIC_n=2_/MIC_n=3_)	
	REO (mg/mL)	FEP (µg/mL)
**ATCC 9027**	80.0 (80.0/40.0/80.0)	1.0 (<0.5/4.0/1.0)
**PS16**	80.0 (80.0/80.0/80.0)	4.0 (1.0/4.0/4.0)
**PT3087**	40.0 (40.0/80.0/40.0)	16.0 (8.0/16.0/16.0)

Note: Illustrative pictures of the AST assays that challenged *P. aeruginosa* PS16 are included in Figure S4 (Supplementary Materials).

**Table 5:** Loaded concentrations of REO and FEP in MLN 3 and MLN 6.

Formulation	FEP Total Dose (µg/mL)	FEP-EE (%)	FEP-LC (µg/mL)	REO-LC (mg/mL)	REO-EE (%)
**MLN 3 (REO)**	-	-	-	42.0	44 ± 3
**MLN 6 (FEP)**	80.0	22 ± 3	17.6	-	-
**MLN 3 (REO-FEP)**	80.0	27 ± 8	21.6	42.0	44 ± 3

**Table 6:** MIC values of REO and FEP as part of loaded formulations and that of the formulation. MIC values are represented as the median of three independent assays.

*P. aeruginosa* Target Strains	Formulation	MIC_median_ (MIC_n=1_/MIC_n=2_/MIC_n=3_) (µL sample/mL)	Active Compound Concentration at MIC Value	
			REO_median_ (n = 1/n = 2/n = 3) (mg/mL)	FEP_median_ (n = 1/n = 2/n = 3) (µg/mL)
ATCC 9027	MLN 6 (empty)	62.5 (500.0/31.3/62.5)	-	-
	MLN 3 (REO)	15.6 (15.6/62.5/15.6)	0.6 (0.6/2.2/0.6)	-
	MLN 6 (FEP)	31.3 (31.3/31.3/15.6)	-	2.5 (2.5/2.5/1.3)
	MLN 3 (REO-FEP)	62.5 (62.5/31.3/62.5)	1.2 (4.4/1.1/2.4)	5.0 (5.0/2.5/5.0)
PS16	MLN 6 (empty)	125.0 (500.0/125.0/125.0)	-	-
	MLN 3 (REO)	31.3 (125.0/31.3/31.3)	1.1 (4.4/1.1/1.1)	-
	MLN 6 (FEP)	31.3 (31.3/31.3/31.3)	-	2.5 (2.5/2.5/2.5)
	MLN 3 (REO-FEP)	125.0 (250.0/125.0/125.0)	4.8 (1.5/4.8/4.8)	10.0 (20.0/10.0/10.0)
PT3087	MLN 6 (empty)	>500.0 (>500.0/>500.0/>500.0)	-	-
	MLN 3 (REO)	125.0 (125.0/125.0/125.0)	4.4 (4.4/4.4/4.4)	-
	MLN 6 (FEP)	250.0 (250.0/250.0/125.0)	-	20.0 (40.0/20.0/10.0)
	MLN 3 (REO– FEP)	500.0 (500.0/500.0/125.0)	19.3 (19.3/19.3/4.8)	40.0 (40.0/40.0/10.0)

Note: Illustrative pictures of the AST assays of MLN 6 (empty) that challenged *P. aeruginosa* PS16 are included in Figure S4 (Supplementary Materials).

## Data Availability

The data presented in this study are contained within the article or supplementary materials.

## Supplementary Materials

The following are available online at https://www.mdpi.com/article/10.3390/antibiotics10111300/s1, Figure S1: Particle size distribution of MLN 5 after preparation, Figure S2 Physical stability of MLN 5 storage for 60 days at 4 °C and protected from light in a glass vial shown as mean particle size values (d(3,2)) and span, Figure S3: Transmission electron microscopy (TEM) images of MLN 3, Figure S4: Illustrative pictures of the AST assay of three sample types (REO, FEP, and MLN 6 (empty)) that challenged *P. aeruginosa* PS16, Figure S5: Effect of sonication time of each of the steps: first sonication (FS); second sonication (SS ) on particle size distribution parameter d(3,2) and span using MLN 5 as reference, Table S1: Effect of FEP concentration in W1 on FEP-EE (%) and FEP-LC for the MNL lipid composition of MNL 3, Table S2: Thermal stability of FEP in aqueous solution determined as the increase in MIC values with respect to that for 1 fresh FEP aliquot and FEP aliquots incubated for 1 week at different temperatures (4 °C, RT (room temperature = 21 °C ± 3 °C), 37 °C, and 42 °C) using *P. aeruginosa* ATCC 27853 as target strain. The results are expressed as the median of the MICs obtained from three independent assays (n = 3), Table S3: Effect of sonication amplitude of each of the steps ( FS- SS) on the particle size distribution parameter d(3,2) and span using MLN 5 as a reference, Table S4: Effect of emulsifier concentrations in the oil (O) and external aqueous phase (W2) on particle size distribution parameters d(3,2) and span using MLN 5 as a reference.
