# Supplementary material for: Essential Oil and Hydrophilic Antibiotic Co-Encapsulation in Multiple Lipid Nanoparticles: Proof of Concept and In Vitro Activity against Pseudomonas aeruginosa
Source: Antibiotics (Basel). 2021 Oct 26;10(11):1300. doi: 10.3390/antibiotics10111300 (PMC8614727; doi:10.3390/antibiotics10111300)
Supplement: Supplementary file 1 [file antibiotics-10-01300-s001.zip › antibiotics-1425170-supplementary.pdf]

Supplementary Materials

Essential Oil and Hydrophilic Antibiotic Co-Encapsulation in Multiple Lipid Nanoparticles: Proof of Concept and In Vitro Activity against *Pseudomonas aeruginosa*

Rayhane Ben-Khalifa, Frédéric Bustos Gaspar, Cristina Pereira, Leila Chekir-Ghedira and Soraya Rodríguez-Rojo

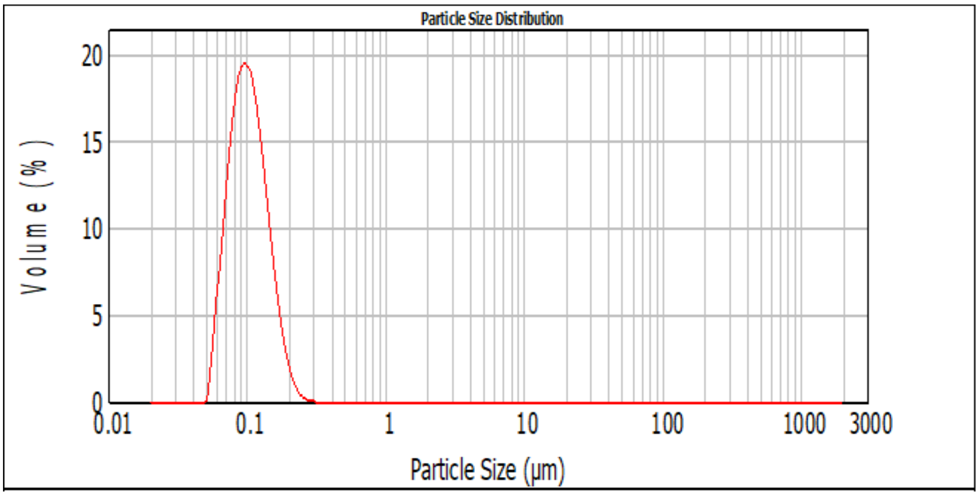

Figure S1. Particle size distribution of MLN 5 after preparation.

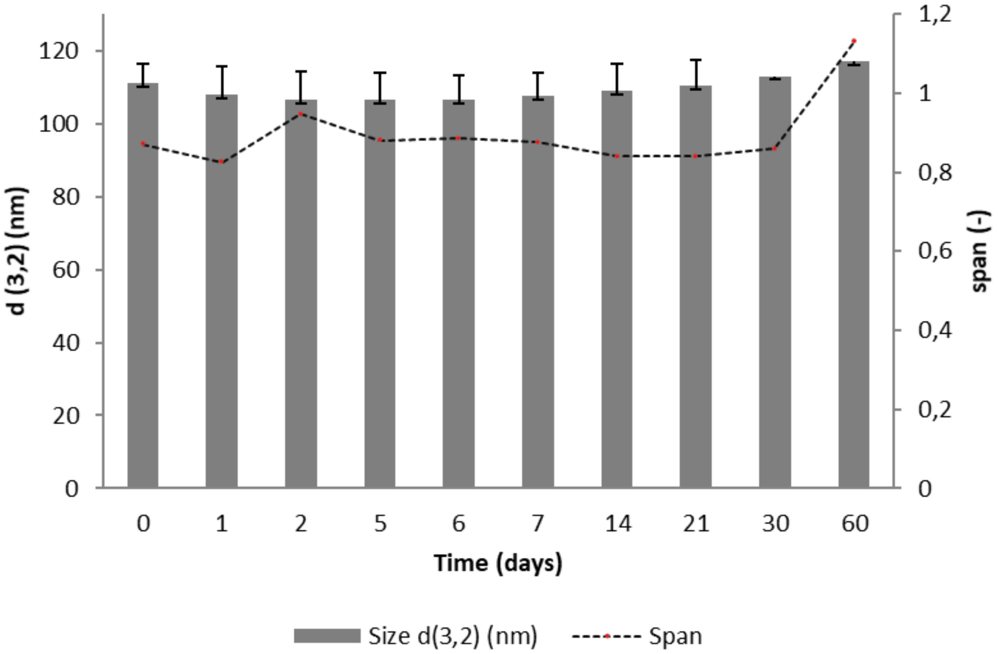

Figure S2. Physical stability of MLN 5 stored for 60 days at 4°C and protected from light in a glass vial shown as mean particle size values (d(3,2)) and span.

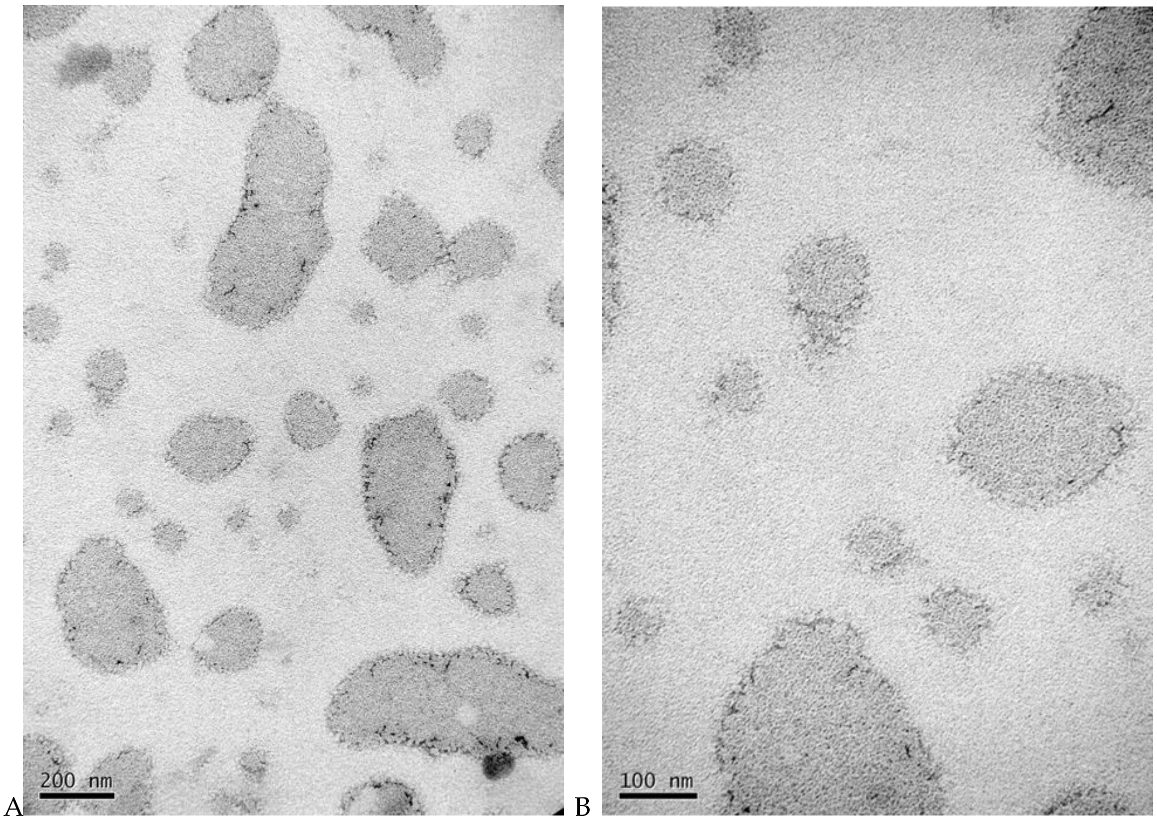

Figure S3: TEM images of MLN 3 loaded with REO and FEP (A): scale 200 nm, (B): scale 100 nm.

Note: Images acquired with a Transmission Electron Microscope (JEOL JEM-1011 HR) at an acceleration voltage of 100 kV from MNL 3 for REO and FEP encapsulation. An adequately diluted sample (300 µL of sample in 2 mL) was deposited onto a TEM mesh grid and air dried afterwards the sample was negatively stained with uranyl acetate. The image shows particles around 100 nm in agreement with the laser diffraction measurements. However, also some deformation and aggregation can be observed (Figure S2. A) due to the thermal deformation of the sample material with the electron beam irradiation of the sample. This effect also affects the internal structure of the nanoparticle. Nevertheless, it could be observed in Figure S2.B that in the external part of the oil phase there are drops, bubbles corresponding to the internal aqueous phase.

Microplate well number	1	2	3	4	5	6	7	8	9	10	11	12
**REO**												
µl of testing sample/mL	500,0	250,0	125,0	62,5	31,3	15,6	7,8	3,9	2,0	1,0	+	-
[REO] (mg/ml)	160,0	80,0	40,0	20,0	10,0	5,0	2,5	1,3	0,6	0,3	+	-
Naked eye observation	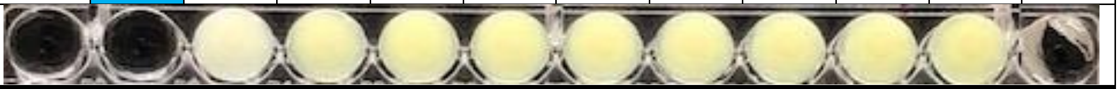											
**FEP**												
µl of testing sample/mL	500,0	250,0	125,0	62,5	31,3	15,6	7,8	3,9	2,0	1,0	+	-
[FEP] (µg/ml)	256,0	128,0	64,0	32,0	16,0	8,0	4,0	2,0	1,0	0,5	+	-
Naked eye observation	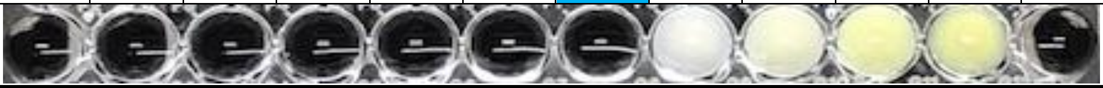											
**MLN 6(empty)**												
µl of testing sample/mL	500,0	250,0	125,0	62,5	31,3	15,6	7,8	3,9	2,0	1,0	+	-
Naked eye observation	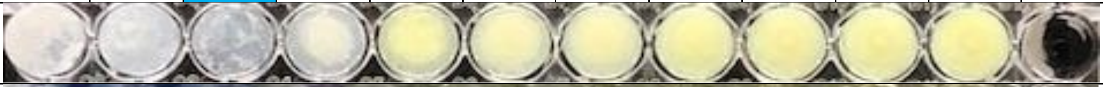											
PrestoBlue	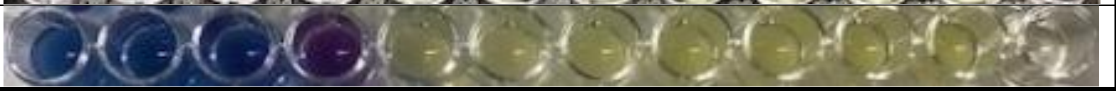											

Figure S4: Illustrative pictures of antimicrobial susceptibility testing (AST) assays that challenged *P. aeruginosa* PS16 with 3 different representative sample types: REO (lipid solution), FEP (aqueous solution), and MLN 6 (empty) (nanoparticle). The

pictures correspond to replicate n=2 and show the ranges of concentrations tested from well number 1 to 10 in the microplate, with wells 11 and 12 corresponding to the positive and the negative controls, respectively.

Note: for the pictures presented, the MIC value for REO (identified in blue) is 250.0 µl of testing sample/mL which corresponds to a concentration of REO of 80.0 mg/mL, the MIC value for FEP (in blue) is 7.8 µl of testing sample/mL which corresponds to a concentration of FEP of 4.0 µg/mL, and the MIC value for MLN 6 (empty) (in blue) is 125.0 µl of testing sample/mL (confirmation of the MIC value performed using the cell viability reagent PrestoBlue on the highest concentrations tested).

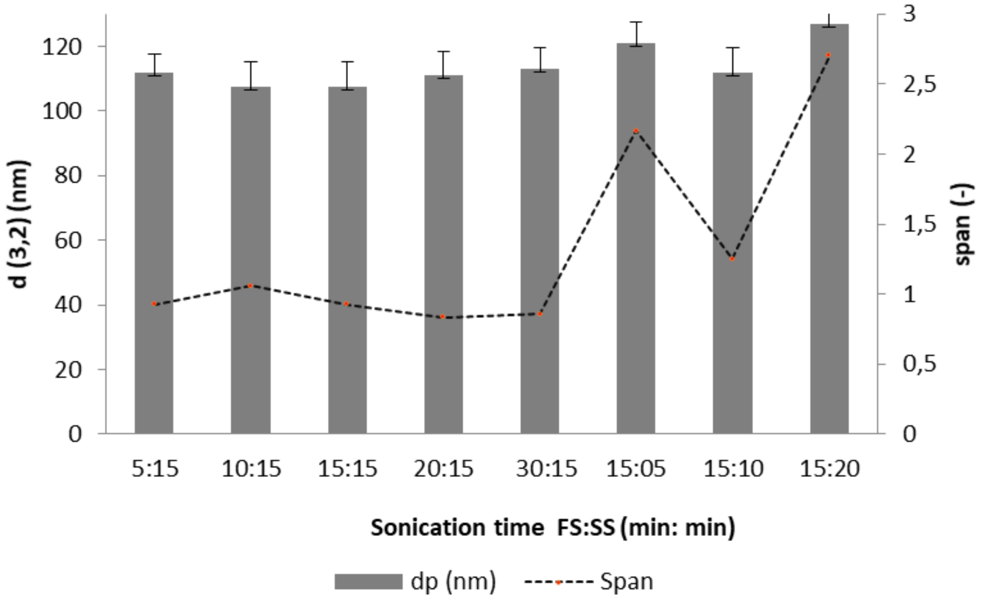

Figure S5. Effect of sonication time of each of the steps: First Sonication (FS): Second sonication (SS) on particle size distribution parameter d(3,2) and span using MLN 5 as reference.

Table S1. Effect of FEP concentration in W1 on FEP-EE (%) and FEP-LC for MNL lipid composition of MNL 3.

Initial concentration (W1) (mg/ ml)	FEP-EE (%)	FEP-LC (µg/ml)
**2**	15	12.0
**10**	27	107.8
**20**	21	168.0
**30**	22	263.8

Table S2. Thermal stability of FEP in aqueous solution determined as the increase in MICs values respect of that for 1 fresh FEP aliquot and FEP aliquots incubated 1 week at different temperatures (4°C, RT (room temperature = 21 ±3°C), 37°C, and 42°C), using *P. aeruginosa* ATCC 27853 as target strain. Results are expressed as the median of the MICs obtained from three independent assays (n=3).

MIC value (µg/ml)					
P. aeruginosa strain	FEP aliquot	FEP aliquot incubated 1 week			
	Freshly thawed	@ 4°C	@ RT	@ 37°C	@ 42°C
ATCC 27853	2	4	4	4	16

FEP stock solution prepared in PBS buffer (pH 6), at a concentration of 512 µg/mL.

Table S3. Effect of sonication amplitude of each of the steps (FS- SS) on particle size distribution parameter d(3,2) and span using MLN 5 as reference.

Sonication Amplitude<br>% FS - % SS	d(3,2) nm	Span
**90% -45%**	223	477
**90% -50%**	153	14
**90% -70%**	115	2
**90% -90%**	111	0.83

Table S4. Effect of emulsifiers concentration in the oil (O) and external aqueous phase (W2) on particle size distribution parameters d(3,2) and span using MLN 5 as reference.

Pluronic ® L64<br>(O)	Tween® 80<br>(W2)	d(3,2)<br>(nm)	Span
**1%**	5%	124±5	3±0.98
**5%**		129±7	5±2
**10%**		112±1	0.80±0.02
**10%**	1%	154±26	10±7
	3%	120±5	3±2
	5%	112±1	0.80±0.02
